# Proton pump inhibitors and the risk of Alzheimer’s disease and non-Alzheimer’s dementias

**DOI:** 10.1038/s41598-020-78199-0

**Published:** 2020-12-03

**Authors:** Francisco Torres-Bondia, Farida Dakterzada, Leonardo Galván, Miquel Buti, Gaston Besanson, Eric Gill, Roman Buil, Jordi de Batlle, Gerard Piñol-Ripoll

**Affiliations:** 1 Unitat Trastorns Cognitius (Cognitive Disorders Unit), Clinical Neuroscience Research Group, Santa Maria University Hospital, IRBLleida, Rovira Roure no. 44, 25198 Lleida, Spain; 2 Pharmacy Department, Servei Català de La Salut (Catalan Health Services), Lleida, Spain; 3 Unitat D’Avaluació Clínica (Clinical Evaluation Unit), Institut Català de La Salut (Catalan Institute of Health), Lleida, Spain; 4 Accenture Innovation Center, Barcelona, Spain; 5 Barcelona Graduate School of Economics (BGSE), Barcelona, Spain; 6 Universitat Oberta de Catalunya (UOC), Barcelona, Spain; 7 Group of Translational Research in Respiratory Medicine, Arnau de Vilanova University Hospital and Santa Maria University Hospital, IRBLleida, Lleida, Spain; 8 Biomedical Research Networking Center in Respiratory Diseases (Centro de Investigación Biomédica en Red de Enfermedades Respiratorias, CIBERES), Madrid, Spain

**Keywords:** Cognitive ageing, Dementia

## Abstract

Proton pump inhibitors (PPIs) are among the most prescribed medications. Previous epidemiological studies have presented contradictory results about PPIs and the risk of dementia. Our objective was to investigate the association between the use of PPIs and an increasing risk of incident AD or non-AD dementias. A community-based retrospective cohort study was conducted based on the data available from 1st January 2002 to 31st December 2015 in the Catalan health service (CatSalut) system. This cohort included all PPI users (N = 36,360) and non-users (N = 99,362). A lag window of 5 years was considered between the beginning of the PPI treatment and the diagnosis of dementia. PPI use was not associated with the risk of AD (adjusted odds ratio (OR) 1.06) (95% CI 0.93–1.21; *p* = 0.408). A weakly but significantly increased risk of non-AD dementias was observed among PPI users (adjusted OR 1.20, 95% CI 1.05–1.37; *p* = 0.007). A higher dose of PPIs was not associated with an increased risk of either AD or non-AD dementias (OR 1.20; 95% CI 0.91–1.61 and OR 0.95; 95% CI 0.74–1.22, respectively). Regarding the number of PPIs used, we observed an increased risk of AD (OR 1.47; 95% CI 1.18–1.83) and non-AD dementias (OR 1.38; 95% CI 1.12–1.70) in users of two types of PPIs compared with those who used only one type. We did not find a higher incidence of AD among PPI users, but a weak increase in the risk of non-AD dementias among PPI users was observed.

## Introduction

Dementia is a syndrome characterized by progressive deterioration of cognitive functions that eventually leads to complete loss of independence in daily life. Alzheimer’s disease (AD) is the most common type of dementia, accounting for 60–80% of cases. Approximately 50 million people have dementia worldwide, and this number is estimated to triple by 2050^1^. Currently, there is no treatment available to slow the progression of cognitive decline in patients living with the disease. Therefore, identifying effective strategies to preventing the onset or slowing the progression of the disease is of great importance. Until now, a wide range of modifiable risk factors for dementia and AD have been identified, including hypertension, diabetes, obesity, depression, and low levels of physical or mental activity^2–8^. The exploration of other potential risk factors is still ongoing and has great importance in searching for the appropriate strategies for reducing AD and dementia incidence.

As a consequence of the increase in life expectancy, multimorbidity and polypharmacy have increased in recent years, especially in the elderly population^9,10^. Polypharmacy is associated with adverse effects such as mortality, falls, adverse drug reactions, and an increase in both hospital stay and readmissions^11,13^. Older individuals are at an even higher risk of adverse effects as a result of decreased kidney and liver function, decreased lean body mass, decreased hearing, vision, cognition, and mobility^14^. Recent studies have shown the relationship between chronic use of different drugs in older people and the development of AD and other dementias. This association appears stronger with benzodiazepines (BZD)^15^, and to a lesser extent, with antidepressants^16^ and anticholinergic drugs^16^.

Some experimental^18^ and clinical^19^ studies in recent years have found that proton pump inhibitors (PPIs) are associated with an increased risk of dementia and AD. PPIs block the H+ and K+-ATPase and, as a result, suppress the secretion of gastric acid. PPIs are indicated for the treatment of gastroesophageal reflux, Barrett’s oesophagus, Zollinger–Ellison syndrome, idiopathic chronic ulcer, and digestive bleeding problems^20^. They are among the most frequently prescribed medications, and the current evidence indicates that 25–70% of PPIs prescriptions have no appropriate indication and lead to the overuse of PPIs^21^. The widespread and long-term use of these medications could have negative consequences on health. One of the most studied potential adverse effects of the long-term use of PPIs is dementia^22^. However, the evidence regarding an association between the use of PPIs and developing dementia is controversial. Three large epidemiological studies—two in Germany and one in Taiwan- showed that long-term exposure to PPIs increases the risk of developing dementia in the elderly^19,23,24^. On the other hand, two other studies, one in Finland and the other in the United States, found no association between the use of PPIs and the risk of incident AD^25,26^. Several systematic review and meta-analysis suggest that there was no association between PPIs use and increased risk of dementia or AD^27–30^.

Machine learning grew up from the idea of making machines (i.e. computers) learn from a wide range of complex, unstructured and semi-structured data. Different machine learning approaches have been developed in the field of cognitive diseases, based on questionnaires data, Magnetic Resonance Imaging data and on other less conventional data sources for the field of cognitive diseases. Although many valuable results have already been achieved in this field, there is still room for improvement. In addition, the use and results of expert systems in machine learning in everyday practice are still unreliable^31–33^.

Because of these discrepancies in the results of the previous studies and the lack of information regarding the use of PPIs and the risk of dementia among Mediterranean populations, we decided to evaluate the association between PPI use and the incidence of AD and non-AD dementias in a retrospective cohort of patients in the Sanitary Region of Lleida (SRL), Spain. We also analysed our data regarding any relationship between age of the patients and dose, type and number of PPIs and incidence of AD and non-AD dementias.

## Methods

### Source of the data

This study is a community-based retrospective cohort study conducted in 2018. It was based on the data available from 1st January 2002 to 31st December 2015 in the Catalan health service (CatSalut) system. This health system provided health coverage to 358,070 inhabitants in the SRL in 2015, which represents 98% of the Lleida county population. The data on PPI consumption were obtained from the number of packages dispensed by the pharmacies. Spain has a public health system where drugs are dispensed in pharmacies after presenting a prescription by a doctor (usually a general practitioner or sometimes a specialist for ambulatory patients). As the data regarding the drug dispensing issued by mutual insurance entities or other insurers, drugs administered to hospitalized patients, and drugs prescribed by private providers who dispensed drugs without a prescription are not reflected in the CatSalut system, they were not included in our study. The clinical and demographic characteristics of the sample and controls were obtained from the data in the Catalan Institute of Health (ICS); this entity is part of the comprehensive public healthcare system of Catalonia (SISCAT).

### Study population

This cohort included all PPI users older than 45 years who had a family physician registered in a basic health area (the basic health area corresponds to a territory and its population which is attended by a primary care team mainly consisting of family physicians, paediatricians, nurses and administrative support staff) of SRL at the beginning of the study.

A minimum lag window of five years between the beginning of the consumption of PPIs and the diagnosis of AD or dementia was considered for the analysis to account for the long latency of AD and dementia development.

All subjects of SRL who filled a prescription for PPI during the period of the study were included in the first database. From this data set the following patients were excluded: (1) patients younger than 45 years, (2) patients diagnosed with AD/dementia at the beginning of the study or during the first five years after the beginning of PPI consumption, (3) patients who passed away or changed their address to outside of RSL during the period of the study, and (4) patients who concomitantly used benzodiazepines or Z-drugs during the study period.

According to these criteria, we detected 36,360 subjects as PPI users who received the medication(s) between the 1st of January 2002 and the 31st of December 2015. We identified 99,362 subjects that had never been treated with these drugs during the same period; therefore, they were enrolled as the control. These non-exposed subjects were recruited from Catalan health service (CatSalut) system and their demographical data were obtained from the data in the Catalan Institute of Health (ICS) as exposed patients.

### Exposure

PPIs were categorized according to the Anatomical Therapeutic Chemical classification system (ATC) as A02BC01 (omeprazole), A02BC02 (pantoprazole), A02BC03 (lansoprazole), A02BC04 (esomeprazole) and A02BC05 (rabeprazole)^34^. All of these PPIs have been approved by the Spanish Agency of Medication and so were included in the study^35^. The use of PPIs was defined as at least one prescription during the study period, and it was evaluated based on the defined daily dose (DDD) accumulated for each subject throughout the study period. Exposure was determined from computerized pharmacy data and consisted of total DDD dispensed to an individual during the period the study. For instance, if a patient consumed IBPs for a while, then stopped consumption, and later restarted IBP use, the total of DDD consumed during the whole time of the study was considered. The DDD is a technical unit of measurement that corresponds to the daily maintenance dose of a drug for its main indication in adults and for a given route of administration. The DDDs of active ingredients are established by the WHO and published on the WHO Collaborating Center for Drug Statistics Methodology website^34^.

Based on the exposure amount, we divided the PPIs users into three groups: (1) very low exposure (< 28 DDDs); (2) low exposure (28–83 DDDs); and (3) high exposure (> 83 DDDs)^24^.

### Variables

Demographic information including age and sex and comorbidities such as hypertension, diabetes mellitus, hyperlipidaemia, stroke, myocardial infarction, depression, anxiety, other affective disorders, sleep disturbances, insomnia and diagnosis of AD or dementia (other than AD) were registered^36^. The diagnosis of dementia was defined as case documentation with one of the following International Statistical Classification of Diseases and Related Health Problems 10th Edition (ICD-10) codes: G30.0, G30.1, G30.8, G30.9, G31.0, G31.01, G31.83, G31.84, G31.85, F01.5, F01.50, F01.51, F02.8, F02.80, F02.81, F03.9, F03.90, or F03.91^36^. Age, sex, hypertension (I10), diabetes (E10, E11, E13) and dyslipidaemia (E78) were considered as a confounding variables.

### Statistical analyses

Participants’ baseline characteristics are described by the number (%) or mean (SD), as appropriate. Logistic regression models were used to estimate the crude and adjusted odds ratios (OR) for the association between the consumption of PPI (ever/never, and according to the consumed dose) and risk of incident AD and non-AD dementias. Similarly, analyses according to specific PPI and the number of different PPIs were also performed. Finally, stratified models by age groups were built. After assessing for potential confounders, all models were adjusted by age, sex, hypertension, diabetes and dyslipidaemia. In addition, time to event (AD and non-AD diagnosis) analyses were also performed, including Kaplan–Meier survival curves and Cox proportional hazard models.

Each set of patients (AD and non-AD) were studied separately using the Scikit-learn package of Python. Each individual group was split into 70% training set and 30% test set. The training dataset was used to create a Random Forest algorithm in order to train it to predict whether a patient will be a sufferer or not. The remaining 30% of patients were used to test the effectiveness of this predictive model. The results shown are the metrics of the predictive model's ability to correctly identify sufferers of each illness. The level of significance was fixed at 0.05. All analyses were performed using Tableau 2019.1 or Stata v12.

### Ethics

The Clinical Investigation Ethical Committee (CEIC P16/109) of IDIAP Jordi Gol approved this study. Due to this is a retrospective cohort study and the patients are blinded for the investigators no written informed consent was obtained according to Clinical Investigation Ethical Committee.

All methods were carried out in accordance with relevant guidelines and regulations.

## Results

**Figure 1:**
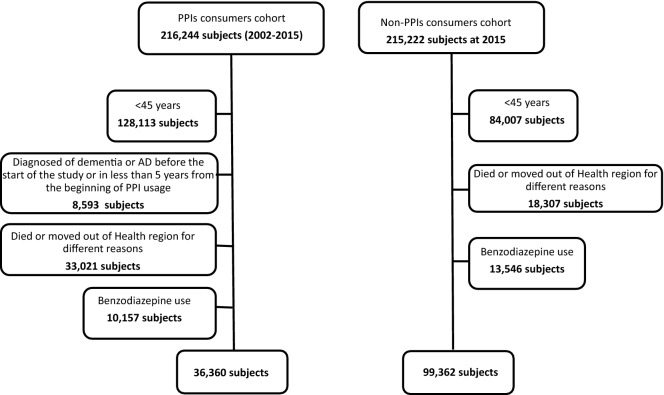
Flowchart of subjects included for the analysis.

We identified 216,224 PPIs users from 1st January 2002 to 31st December 2015. From this population of PPI users, 179,884 persons were excluded. Of those, 128,113 were excluded because they were younger than 45 at the beginning of the study, 8,593 were excluded because they were diagnosed with dementia or AD before the start of the study or in less than 5 years from the beginning of PPI usage, 33,021 were excluded because they died or moved out of SRL for different reasons during the period of the study, and 10,157 were excluded because of benzodiazepine use (Fig. 1). Finally, 36,360 patients were included for the analysis. The non-exposed persons were 99,362 who had never used PPIs coming from the community. There was no significant difference between PPI users and non-users regarding age. Nevertheless, other demographic characteristics and the prevalence of comorbidities were significantly different between the two groups (Table 1).

**Table 1:** Characteristics of the study population according to the consumption of proton pump inhibitors (PPIs).

	Non-PPIs users (N = 99,362)	PPIs users (N = 36,360)	p-value
Age, mean (SD)	66.8 (13.2)	66.9 (11.8)	0.308
Women, n (%)	47,755 (48.1%)	14,522 (39.9%)	< 0.001
Hypertension, n (%)	17,930 (18.1%)	18,180 (50%)	< 0.001
Diabetes, n (%)	6986 (7%)	7835 (21.6%)	< 0.001
Dislipemia, n (%)	14,597 (14.7%)	15,175 (41.7%)	< 0.001
Depression, n (%)	3125 (3.2%)	1543 (4.2%)	< 0.001
Anxiety, n (%)	4941 (5%)	2233 (6.1%)	< 0.001
Sleep disturbances, n (%)	1149 (1.2%)	1266 (3.5%)	< 0.001
Affective disorders, n (%)	358 (0.4%)	257 (0.7%)	< 0.001
Cardiopathy, n (%)	324 (0.3%)	1395 (3.8%)	< 0.001
Alzheimer disease, n (%)	696 (0.7%)	429 (1.2%)	< 0.001
Other dementias, n (%)	645 (0.7%)	490 (1.4%)	< 0.001

During the study period we detected 429 (1.2%) subjects with AD among the PPI users and 696 (0.7%) among the non-users. On the other hand, among the PPI users 490 (1.4%) patients developed other types of dementia versus 645 (0.7%) patients among the PPI non-users (Table 1).

In this study, omeprazole was the most commonly used PPI (73.9%) followed by lansoprazole (10.2%), pantoprazole (8.2%), rabeprazole (4.1%), and esomeprazole (3.7%). We analysed the number of PPIs used with and without diagnosis of all types of dementia. Among individuals without any type of dementia, 76.3% used only one type of PPI, 18.6% used two types, 4.1% used three types, 0.8% used four types, and 0.1% used five types. Meanwhile, among patients with the diagnosis of all types of dementia 64.5% received one type of PPI, 28.3% received two types, 6.1% received three types, and 1.1% received four types. Regarding sex, women used significantly higher doses of PPIs (higher DDD) compared with men [586.6 (8.8) vs 548.9 (6.8), p < 0.001]. However, when comparing each medication individually, this sex difference was only significant for omeprazole [541.1 (8.6) DDD for women vs 505.3 (6.6) DDD for men, p < 0.001].

To evaluate the relative risk induced by PPIs, we analysed the risk of incident AD and non-AD dementias in PPI users compared with the control population. We found that the risk of both incident AD [odds ratio (OR) 1.69; 95% CI 1.50–1.91; p < 0.001] and non-AD dementias (OR 2.09; 95% CI 1.86–2.35; p < 0.001) was higher among PPIs users. After adjusting the data for age, sex, hypertension, diabetes, and dyslipidaemia, the OR was 1.06 (95% CI 0.93–1.21; p = 0.408) for AD and 1.20 (95% CI 1.05–1.37; p = 0.007) for non-AD dementias.

To examine the dose–response relationship, we analysed the risk of incident AD and non-AD dementias between groups divided by exposure dose. We detected a dose–response relationship regarding risk of AD, as the patients who received 28–83 DDD and > 83 DDD demonstrated a higher risk of incident AD (OR 1.50; 95% CI 1.05–2.15 and OR 2.49; 95% CI 1.90–3.30, respectively) compared with those who received < 28 DDD (Table 2). However, this association was no longer significant after adjusting the data for confounding factors (Table 2). Regarding the risk of non-AD dementias, there was no suggestion of a dose–response relationship either as the higher risk observed in > 83 DDD compared with < 28 DDD was abolished after data adjustment for the covariates (Table 3).

**Table 2:** PPI consumption and risk of AD according to dose and age groups.

	Unadjusted OR (95% CI)	Adjusted* OR (95% CI)
**All**		
Dose consumed		
< 28 DDD	Ref.	Ref.
28-83 DDD	1.50 (1.05-2.15)	1.27 (0.88-1.83)
> 83 DDD	2.49 (1.90-3.30)	1.20 (0.91-1.61)
**< 65 years**		
Dose consumed		
< 28 DDD	Ref.	Ref.
28-83 DDD	1.50 (1.05-2.15)	0.40 (0.02-2.73)
> 83 DDD	2.49 (1.90-3.30)	NA**
**65-80 years**		
Dose consumed		
< 28 DDD	Ref.	Ref.
28-83 DDD	0.99 (0.54-1.79)	0.93 (0.50-1.70)
> 83 DDD	1.37 (0.90-2.16)	1.08 (0.71-1.72)
**> 80 years**		
Dose consumed		
< 28 DDD	Ref.	Ref.
28-83 DDD	1.50 (0.93-2.43)	1.52 (0.94-2.46)
> 83 DDD	1.17 (0.81-1.74)	1.19 (0.82-1.77)

*CI* confidence interval, *OR* odds ratio, *PPI* proton pump inhibitor.

*Adjusted by age, sex, hypertension, diabetes, dyslipidaemia.

** < 28 DDD (N = 3,075), 29–83 DDD (N = 13,183), > 83 DDD (N = 20,102).

*** < 65 years old (N = 68,785), 65–80 years old (N = 42,067), > 80 years old (N = 24,870).

**Table 3:** PPI consumption and risk of non-AD dementias according to dose and age groups.

	Unadjusted OR (95% CI)	Adjusted* OR (95% CI)
All		
Dose consumed		
< 28 DDD	Ref.	Ref.
28–83 DDD	0.89 (0.62–1.28)	0.72 (0.50–1.04)
> 83 DDD	2.24 (1.76–2.87)	0.95 (0.74–1.22)
< 65 years		
Dose consumed		
< 28 DDD	Ref.	Ref.
28–83 DDD	NA**	NA**
> 83 DDD	3.43 (0.51–67.09)	2.32 (0.33–45.88)
65–80 years		
Dose consumed		
< 28 DDD	Ref.	Ref.
28–83 DDD	0.83 (0.41–1.60)	0.78 (0.39–1.53)
> 83 DDD	1.32 (0.84–2.15)	1.02 (0.65–1.67)
> 80 years		
Dose consumed		
< 28 DDD	Ref.	Ref.
28–83 DDD	0.67 (0.42–1.02)	0.67 (0.43–1.04)
> 83 DDD	0.86 (0.64–1.16)	0.83 (0.62–1.13)

*CI* confidence interval, *OR* odds ratio, *PPI* proton pump inhibitor.

*Adjusted by age, sex, hypertension, diabetes, dyslipidaemia.

** < 28 DDD (N = 3,075), 29–83 DDD (N = 13,183), > 83 DDD (N = 20,102).

*** < 65 years old (N = 68,785), 65–80 years old (N = 42,067), > 80 years old (N = 24,870).

To evaluate the effect of exposure dose in relation to age, we divided the study population into three age groups: 45–65, 65–80, and > 80 years old. After comparing patients who used higher doses of PPIs with the reference group, < 28 DDD, we did not detect a higher risk of AD or non-AD dementias in any of the age groups after data adjustment (Tables 2 and 3).

Regarding the type of PPI used, esomeprazole and pantoprazole showed a higher risk of AD and non-AD dementias. Esomeprazole had an OR of 1.47 (95% CI 1.10–2.13; p = 0.026) for AD and an OR of 1.40 (95% CI 0.99–1.99; p = 0.056) for non-AD dementias. In the case of pantoprazole, the OR was 1.35 (95% CI 1.04–2.14; p = 0.027) for AD and 1.36 (95% CI 1.06–1.74; p = 0.017) for non-AD dementias (Table 4).

**Table 4:** Association between specific PPIs (compared with PPI non-users) and risk of AD and non-AD dementias.

	AD unadjusted OR (95% CI)	p-value	AD adjusted OR (95% CI)*	p-value	Non-AD dementias unadjusted OR (95% CI)	p-value	Non-AD dementias adjusted OR (95% CI)*	p-value
Omeprazole	0.97 (0.61–1.52)	0.877	0.71 (0.45–1.13)	0.146	1.06 (0.68–1.64)	0.812	0.74 (0.47–1.17)	0.203
Pantoprazole	1.64 (1.27–2.13)	< 0.001	1.35 (1.04–1.76)	0.027	1.68 (1.32–2.14)	< 0.001	1.36 (1.06–1.74)	0.017
Lansoprazole	1.12 (0.85–1.46)	0.436	0.98 (0.74–1.29)	0.865	1.22 (0.95–1.56)	0.120	1.07 (0.83–1.37)	0.624
Rabeprazole	1.49 (1.04–2.14)	0.031	1.30 (0.90–1.88)	0.168	1.29 (0.90–1.85)	0.168	1.11 (0.76–1.60)	0.599
Esomeprazole	1.75 (1.23–2.49)	0.002	1.47 (1.10–2.13)	0.036	1.66 (1.18–2.32)	0.004	1.40 (0.99–1.99)	0.056
Combination	1.45 (1.35–1.55)	< 0.001	1.09 (1.01–1.18)	0.036	1.58 (1.49–1.69)	< 0.001	1.15 (1.06–1.24)	< 0.001

*CI* confidence interval, *OR* odds ratio, *PPI* proton pump inhibitor.

*Adjusted by age, sex, hypertension, diabetes, dyslipidaemia.

Regarding the number of PPIs used, we compared the patients who consumed only one type of PPI with patients who took more than one type of PPI. We observed an increased relative risk of AD (OR 1.47; 95% CI 1.18–1.83) and non-AD dementias (OR 1.38; 95% CI 1.12–1.70) in users of two types of PPIs compared with those who consumed only one type of PPI. We did not detect a higher risk of either AD or non-AD dementias among those who consumed more than two types of PPIs (Suppl. Table 1).

The results of time to event analyses were very similar and can be found in the supplementary material (Suppl. Tables 2, 3 and Fig. 1).

To evaluate which variables of our sample were more relevant to identifying patients diagnosed with dementia, we present a random forest tree. Each individual group of patients (AD and non-AD) were split into a training and test set in the following way; 70% of each group was used to train the model. These were the cases that were fed to the Random Forest algorithm in order for the model to be trained in identifying the characteristics which in turn allow us to predict if a patient will be a sufferer or not. The remaining 30% of patients were used to test the effectiveness of this predictive model. The results of this analysis revealed that age, HTA and several variables related to PPI were the most relevant variables for identifying subjects diagnosed with AD and non-AD dementias. (Table 5).

**Table 5:** Variable importance scores in the random forest tree for AD and non-AD population.

Alzheimer's disease		Non-AD dementia	
Features	Coefficient	Features	Coefficient
Age	0.482716	Age	0.485464
Omeprazol	0.088696	Hypertension	0.121935
Hypertension	0.068832	Omeprazol	0.094254
PPI	0.068038	PPI	0.080469
Esomeprazol	0.020370	Pantoprazol	0.010879
Pantoprazol	0.008563	Lansoprazol	0.007852
Diabetes	0.002449	Esomeprazol	0.006407
Lansoprazol	0.002116	Diabetes	0.002871

## Discussion

We evaluated the risk of PPI use and the incidence of AD and other types of dementia in a community-based retrospective cohort. We considered at least a five-year lag window between the start of PPI usage and the incidence of any type of dementia. Our data analysis demonstrated no association between the use of PPIs and incidence of AD and a discretely increased risk of non-AD dementias after adjusting for comorbidities.

Detecting risk factors for dementia is a fundamental step for its prevention. Age is one of the most important risk factors for dementia. Therefore, as the global population of the world ages, the prevalence of dementia and especially AD is increasing^37^. Normally, the presence of health problems grows in the elderly, and as a result polypharmacy is common among this population. The medications used are often of special interest as a risk factor for dementia. Therefore, during recent years, the association between the long-term use of some of these medications, including PPIs, and dementia has been examined in some studies^19,23–26^. The existing evidence regarding the use of PPIs and increased risk of AD or other types of dementia is limited and inconsistent.

Dementia and in particular AD have a long preclinical and prodromal period of approximately 10–15 years. Among the previous studies, the study by Taipale et al. had a more similar design to ours. They considered a lag window of 5 years (it was defined as no inclusion of the PPI use during the 5-year period before the AD diagnosis in the analyses) and evaluated the risk of AD in particular. Their results demonstrated no risk of incident AD after PPI use and applying a five-year lag (OR 1.05; 95% CI 1.03–1.07)^10^. Although the window period we defined was not exactly the same as in the study by Taipale et al., we did not observe any association between the use of PPIs and the risk of AD among PPI users (OR 1.06; 95% CI 0.93—1.21) either. In the study by Taipale et al., the risk of incident AD was assessed with and without applying the lag window. Some associations that they found between higher doses of consumption and increased risk of AD in the model without the lag window lost their significance in the models with the lag window^25^. This emphasizes the importance of taking an adequate lag window when considering these types of studies. In a prospective cohort study by Gray et al., a similar result was also reported, and they did not find any association between PPI use and risk of dementia or AD (HR 1.13; 95% CI 0.82–1.56 for highest dosis)^26^.

Unlike the aforementioned studies, Gomm et al. and Tai et al. found a higher risk of dementia among PPI users (HR 1.44; 95% CI 1.36–1.52 and OR 1.22; 95% CI 1.05–1.42, respectively)^19,24^. These different results can be attributed to their considerable differences in study design. In these two studies, among the other differences, no lag time between the use of PPIs and incidence of dementia was considered. Moreover, neither study specified the type of dementia.

Comparing our results with several meta-analysis or systematic review, only Zhang et al. showed an increased dementia risk with PPIs use (HR 1.29; 95% CI 1.12–1.49). In subgroup analyses, a significant association was detected between PPI use and the risk of dementia in Europe (HR 1.46; 95% CI 1.23–1.73) and among participants aged ≥ 65 years (HR 1.39, 95% CI 1.17–1.65). For the factor follow-up time ≥ 5 years, the pooled HR was 1.28 (95% CI 1.12–1.46), demonstrating a 1.28-fold increase in the risk of dementia among PPIs users^29^. However, other studies did not found this increased risk of dementia (RR 1.23; 95% CI 0.90–1.67), (RR 1.05; 95% CI 0.96–1.15) and (HR 1.10; 95 CI 0.88–1.37), respectively^28–30^. This risk was also not observed when we specifically assessed the risk of AD RR 1.01 (95% CI 0.78–1.32) and HR 1.06 (95% CI 0.72–1.55) respectively^27,30^.

Although dementia includes a set of diseases with shared clinical manifestations, especially in their advanced stages, the underlying biological and molecular mechanisms are different among them. Thus, for example, AD is caused by the deposit of β-amyloid and hyperphosphorylated tau in brain of the patients, while in frontotemporal lobar degeneration deposits of tau or TDP-43 can be characterized or Lewy body dementia is characterized by the presence of alpha-synuclein deposits^38–41^. Some symptoms may be shared by some of them in the very early and final stages of the disease, making the differential diagnosis difficult. However, the causes of neurodegeneration are very different in each one of them. As a result, evaluating the risk of PPIs on dementia as a whole may mask their effect on a particular type of dementia.

In our study, we evaluated the risk of incident AD in particular and non-AD dementias as a whole. Non-AD dementias are not as prevalent as AD, and therefore we had a limited number of patients for each type of non-AD dementia. This limitation made us evaluate the risk of non-AD dementias jointly. We detected an increased risk of non-AD dementias in PPI users compared with non-users. However, no dose–response relationship was found after comparing higher doses with the low dose of PPI consumption. We cannot exclude the possibility that the presence of some confounding factors, such as the APOE4 allele, alcoholism or smoking, BMI, ostheoporosis or polypharmacy that were not used for the data adjustment influenced this result. Another possibility is that analysing the various types of non-AD dementias together and omitting the different pathophysiological processes behind each may have resulted this discrepancy in outcome.

We also evaluated the risk of dementia in different age groups. Gomm et al. found a reduction in dementia risk with PPI use with increased age (HR 1.31; 95% CI 1.22–1.43). Although not significant, we also observed a risk reduction tendency in in elderly subjects^19^.

In accordance with the other studies, omeprazole was the most commonly used PPI in our study population. We did not observe a higher risk of dementia regarding use of this medication, whereas pantoprazole and esomeprazole increased this risk. However, the percentage of the subjects using these two medications was very low. Taipale and collaborators did not find any association between the types of PPI and the risk of AD except a weak association with lansoprazole too (OR 1.06; 95% CI 1.02–1.10)^24,25^. Although in the random forest tree the consumption of omeprazole and PPIs in general can help identify population groups with AD or other dementias, this does not mean that the risk is induced by the drugs themselves.

This study has some strengths including the long follow-up period which allows us to analyse our data with a five-year lag window between exposure and outcome to reduce the possibility of selection bias. There is considerable evidence associating the risk of incident dementia with the use of benzodiazepines^42,43^. The use of these medications was considered as a confounding variable in none of the previous studies. In our study, to avoid the risk of confounding bias regarding the use of benzodiazepines, we excluded all individuals who received these drugs at any doses. The drugs dispensed by the pharmacies were used as a source of the data on drug use instead of drug prescriptions to avoid the primary nonadherence problem. The Catalan health service is a public system that covers all citizens regardless of their socioeconomic situation; therefore, our study population can be considered a representative sample of the country’s population. Our study also had some limitations. The diagnosis of dementia and AD was assessed based on the records in the claims data according ICD codes and it was not verified. Our study period was from 2003 to 2015. The diagnosis criteria for AD was modified during this period of time and surely affected the neurologist’s judgement at the time of diagnosis. Our limited number of patients with different types of non-AD dementias prevented us from evaluating the association between use of PPIs and each type of dementia in particular. We considered a five-year lag window as an inclusion criterion. This length of time can be considered short compared with the long preclinical and prodromal period of the disease; however, considering a longer lag window in our study was impossible because it would dramatically reduce the number of individuals included in the study.

In conclusion, we found that the incidence of AD was not higher among PPI users, and a slight increase in the risk of non-AD dementia was observed. As the consumption of PPIs is a useful variable for identifying patients with dementia according to the random forest tree, presence of some chronic and co-morbid pathologies and the resulted polypharmacy, including the increased consumption of PPIs, probably give rise to the increased risk of dementia observed in previous studies.

## Supplementary Information

Supplementary Information
