# Supplementary material for: Proton pump inhibitors and the risk of Alzheimer’s disease and non-Alzheimer’s dementias
Source: Sci Rep. 2020 Dec 3;10:21046. doi: 10.1038/s41598-020-78199-0 (PMC7713356; doi:10.1038/s41598-020-78199-0)
Supplement: Supplementary file 1 — Supplementary Information [file 41598_2020_78199_MOESM1_ESM.docx]

**Proton pump inhibitors and the risk of Alzheimer’s disease and non-Alzheimer’s dementias**

Francisco Torres-Bondia^1*^, Farida Dakterzada^2*^, Leonardo Galván^3^, Miquel Buti^4^, Gaston Besanson^5,6^, Eric Gill^5^, Roman Buil^5,7^, Jordi de Batlle^8,9**^, Gerard Piñol-Ripoll^2**^

(1) Pharmacy Department, Clinical Neuroscience Research group, IRBLleida, Arnau de Vilanova University Hospital, Lleida, Spain.

(2) Unitat Trastorns Cognitius (Cognitive Disorders Unit), Clinical Neuroscience Research group, Santa Maria University Hospital, IRBLleida, Lleida, Spain.

(3) Pharmacy Department, Servei Català de la Salut (Catalan Health Services), Lleida, Spain.

(4) Unitat d'Avaluació Clínica (Clinical Evaluation Unit), Institut Català de la Salut (Catalan Institute of Health), Lleida, Spain.

(5) Accenture Innovation Center, Barcelona, Spain.

(6) Barcelona Graduate School of Economics (BGSE), Barcelona, Spain.

(7) Universitat Oberta de Catalunya (UOC), Barcelona, Spain

(8) Group of Translational Research in Respiratory Medicine, Arnau de Vilanova University Hospital and Santa Maria University Hospital, IRBLleida, Lleida, Spain.

(9) Biomedical Research Networking Center in Respiratory Diseases (Centro de Investigación Biomédica en Red de Enfermedades Respiratorias, CIBERES), Madrid, Spain.

* Co-first authors. FT-B and FD contributed equally to this study.

**Co-corresponding author

****Corresponding author:**

Gerard Piñol Ripoll

Cognitive Disorders Unit

Hospital Universitari Santa Maria

Rovira Roure nº 44, 25198, Lleida, Spain

Telephone: 34-937-727222 Ext. 173. Fax: 34-976-727366

E-mail: gerard_437302@hotmail.com

**Suppl. Table 1.** Association between number of PPIs used and risk of AD and non-AD dementias. Adjusted by age, sex, hypertension, diabetes, dyslipemia.

**Suppl. Figure 1.** Kaplan–Meier survival curves of time to Alzheimer (panels A & B) and non-Alzheimer dementia (panels C & D) diagnosis according to PPI consumption. Panels B & D have a magnified Y-axis scale.

**
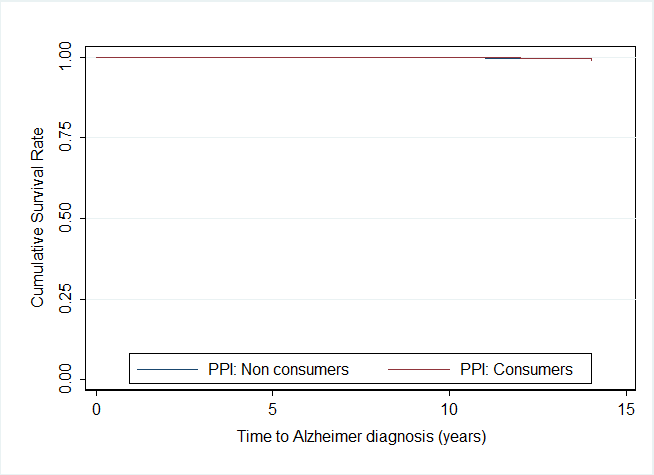

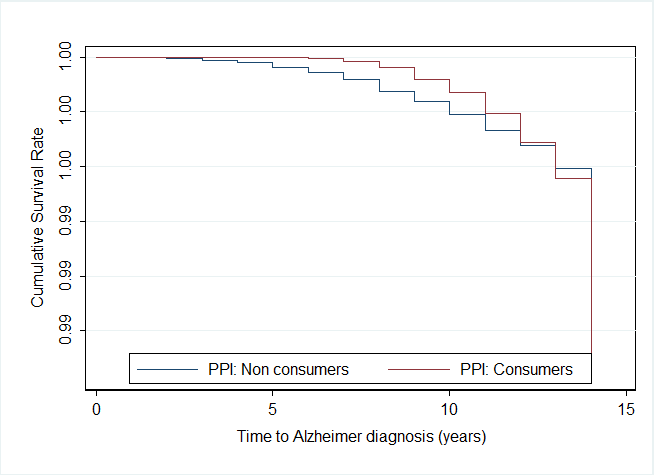

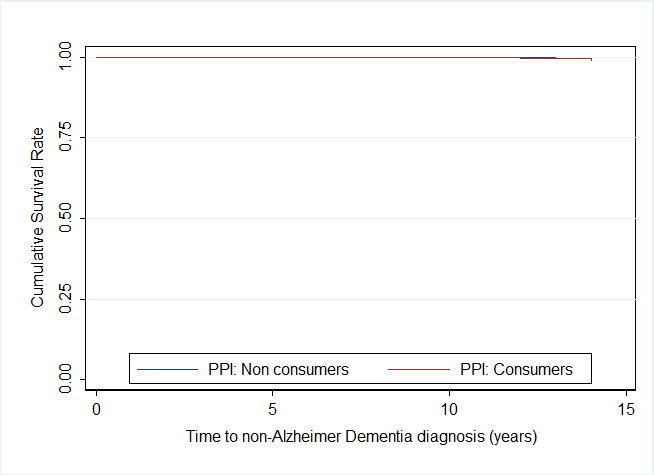

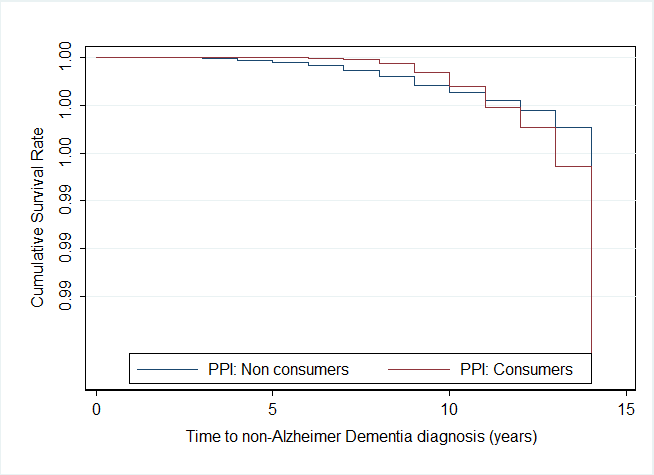
**

D

C

B

A

| **Suppl. Table 2.** Crude and adjusted association between PPI consumption and risk of Alzheimer's disease diagnosis, according to age groups, in 135.722 subjects with a mean follow-up of 14 years. | | |
| --- | --- | --- |
|  | Unadjusted HR (95% CI) | Adjusted* HR (95% CI) |
| All |  |  |
| Dose consumed |  |  |
| <28 DDD | ref | ref |
| 28-83 DDD | 0.90 (0.58-1.39) | 0.78 (0.50-1.21) |
| >83 DDD | 1.89 (1.26-2.84) | 0.87 (0.58-1.32) |
| <65 years |  |  |
| Dose consumed |  |  |
| <28 DDD | ref | ref |
| 28-83 DDD | 0.38 (0.06-2.29) | 0.36 (0.06-2.19) |
| >83 DDD | NA** | NA** |
| 65-80 years |  |  |
| Dose consumed |  |  |
| <28 DDD | Ref | ref |
| 28-83 DDD | 0.91 (0.42-1.96) | 0.89 (0.41-1.91) |
| >83 DDD | 1.27 (0.62-2.60) | 1.01 (0.49-2.08) |
| >80 years |  |  |
| Dose consumed |  |  |
| <28 DDD | Ref | ref |
| 28-83 DDD | 0.72 (0.41-1.28) | 0.72 (0.41-1.27) |
| >83 DDD | 0.73 (0.43-1.23) | 0.73 (0.43-1.24) |
| *Adjusted by age, sex, hypertension, diabetes, dyslipemia. ** Cells shown as NA when the number of events for the given cell was less than 5. | | |
| **Suppl. Table 3.** Crude and adjusted association between PPI consumption and risk of non- Alzheimer's disease dementia diagnosis, according to age groups, in 135.722 subjects with a mean follow-up of 14 years. | | |
|  | Unadjusted HR (95% CI) | Adjusted* HR (95% CI) |
| All |  |  |
| Dose consumed |  |  |
| <28 DDD | ref | ref |
| 28-83 DDD | 0.75 (0.50-1.13) | 0.63 (0.42-0.95) |
| >83 DDD | 1.85 (1.27-2.69) | 0.74 (0.51-1.08) |
| <65 years |  |  |
| Dose consumed |  |  |
| <28 DDD | ref | ref |
| 28-83 DDD | NA** | NA** |
| >83 DDD | NA** | NA** |
| 65-80 years |  |  |
| Dose consumed |  |  |
| <28 DDD | ref | ref |
| 28-83 DDD | 0.61 (0.29-1.29) | 0.59 (0.28-1.25) |
| >83 DDD | 0.95 (0.48-1.89) | 0.74 (0.37-1.47) |
| >80 years |  |  |
| Dose consumed |  |  |
| <28 DDD | ref | Ref |
| 28-83 DDD | 0.62 (0.38-1.00) | 0.62 (0.38-1.00) |
| >83 DDD | 0.69 (0.44-1.07) | 0.66 (0.43-1.04) |
| *Adjusted by age, sex, hypertension, diabetes, dyslipemia. ** Cells shown as NA when the number of events for the given cell was less than 5. | | |
